# Suppression of a Novel Vitellogenesis-Inhibiting Hormone Significantly Increases Ovarian Vitellogenesis in the Black Tiger Shrimp, *Penaeus monodon*

**DOI:** 10.3389/fendo.2021.760538

**Published:** 2021-11-08

**Authors:** Phaivit Laphyai, Thanapong Kruangkum, Charoonroj Chotwiwatthanakun, Wanita Semchuchot, Prawporn Thaijongrak, Prasert Sobhon, Pei-San Tsai, Rapeepun Vanichviriyakit

**Affiliations:** ^1^ Center of Excellence for Shrimp Molecular Biology and Biotechnology (Centex Shrimp), Faculty of Science, Mahidol University, Bangkok, Thailand; ^2^ Department of Anatomy, Faculty of Science, Mahidol University, Bangkok, Thailand; ^3^ Academic and Curriculum Division, Nakhonsawan Campus, Mahidol University, Nakhonsawan, Thailand; ^4^ Department of Science, Faculty of Science and Technology, Prince of Songkla University, Pattani, Thailand; ^5^ Department of Clinical Sciences and Public Health, Faculty of Veterinary Science, Mahidol University, Nakhonpathom, Thailand; ^6^ Department of Integrative Physiology, University of Colorado, Boulder, CO, United States

**Keywords:** crustacean hyperglycemic hormone, vitellogenin, neurohormone, crustacean, reproduction

## Abstract

In this study, a novel Crustacean Hyperglycemic Hormone-type II gene *(CHH-type II)* was identified and biologically characterized in a shrimp, *Penaeus monodon*. Based on its structure and function, this gene was named *P. monodon vitellogenesis-inhibiting hormone* (*PemVIH*). The complete cDNA sequence of *PemVIH* consisted of 1,022 nt with an open reading frame (ORF) of 339 nt encoding a polypeptide of 112 amino acids. It was classified as a member of the CHH-type II family based on conserved cysteine residues, a characteristically positioned glycine residue, and the absence of CHH precursor-related peptide (CPRP) domain. The deduced mature PemVIH shared the highest sequence similarities with giant river prawn sinus gland peptide A. Unlike *P. monodon gonad-inhibiting hormone* (*PemGIH*), *PemVIH* was expressed only in the brain and ventral nerve cord, but not the eyestalks. Whole mount immunofluorescence using a newly generated PemVIH antiserum detected positive signals in neuronal cluster 9/11 and 17 of the brain, commissural ganglion (CoG), and neuronal clusters of ventral nerve cord. The presence of PemVIH-positive neurons in CoG, a part of stomatogastric nervous system, suggested a potential mechanism for crosstalk between nutritional and reproductive signaling. The role of *PemVIH* in vitellogenesis was evaluated using RNA interference technique. Temporal knockdown of *PemVIH* in female subadults resulted in a 3-fold increase in ovarian vitellogenin expression, suggesting an inhibitory role of *PemVIH* in vitellogenesis. This study provided novel insight into the control of vitellogenesis and additional strategies for improving ovarian maturation in *P. monodon* without the current harmful practice of eyestalk ablation.

## Introduction

Hatchery production of the black tiger shrimp, *Penaeus monodon*, is limited by the success and efficiency in female broodstock manipulation (1). A period of up to a month is required for ovarian maturation from breeding to spawning. Attempts to shorten this maturation period have traditionally relied on the suppression of gonad-inhibiting hormone (GIH) using unilateral eyestalk ablation (2, 3). This technique is known to stimulate ovarian maturation by eliminating GIH from eyestalks. However, unilateral eyestalk ablation is associated with a decrease in female broodstock fecundity and offspring quality (4–6). As such, injections of double stranded (ds) RNA or antibody against *P. monodon* GIH (PemGIH) into female *P. monodon* broodstock have been introduced as alternatives to accelerate ovarian maturation. Although both techniques have been met with some success in enhancing ovarian stage development and spawning, they failed to achieve the efficiency of eyestalk ablation (7, 8).

GIH is a neurohormone in the family of crustacean hyperglycemic hormone-type II (CHH-type II). This family also includes other essential inhibiting hormones including molt-inhibiting hormone (MIH), mandibular organ-inhibiting hormone (MOIH), and vitellogenesis-inhibiting hormone (VIH) (9–11). GIH and VIH have previously been recognized as the same hormone in several species (12, 13). However, as the names imply, GIH broadly inhibits gonadal maturation, whereas VIH has a more specific role in inhibiting the production of vitellogenin (Vg), a major yolk protein, in hepatopancreas (HP) and ovary, which may in turn inhibit ovarian maturation in several crustaceans (12, 14–16). In this sense, multiple inhibitory hormones may assume seemingly overlapping functions to inhibit crustacean ovarian maturation. The presence of multiple peptide hormones acting as ovarian inhibitors has been reported in most crustacean species examined (10, 17–19). However, in *P. monodon*, the only gonadal inhibitor identified since 2008 was *PemGIH* (20). This necessitated a search for additional *P. monodon* gonadal inhibitory hormones in order to improve the reproduction of this commercially important shrimp.

In this study, we identified a novel CHH-type II gene in *P. monodon*, named *P. monodon vitellogenesis-inhibiting hormone* (*PemVIH*). The complete *PemVIH* cDNA sequence was obtained from our transcriptomic data of the central nervous tissues and characterized by bioinformatics analyses. We showed that *PemVIH* is expressed exclusively in nervous tissues of female subadults and broodstock, but not in the eyestalk. Using a newly generated PemVIH-specific antiserum, we localized PemVIH to various parts of the nervous system, particularly in circumesophageal commissural ganglia. In addition, the temporal knockdown of *PemVIH* significantly increased ovarian vitellogenesis of subadult females, demonstrating that PemVIH is a bona fide vitellogenesis inhibitor that can be manipulated to enhance the reproduction of female *P. monodon*.

## Materials and Methods

### Animal Ethics and Tissue Harvest for RNA Isolation

*P. monodon* female broodstock in previtellogenic stage (80-85 grams body weight, n = 5) and unilateral eyestalk-ablated female broodstock (at day 5 after eyestalk ablation, n = 3) were obtained from Shrimp Genetic Improvement Center, Suratthani, Thailand. Subadult female shrimp (20-30 grams body weight, in premolt stage, n = 30) were obtained from commercial farms at Pathumthani, Thailand. All shrimp were checked for pathogens, including White spot syndrome virus (WSSV), Yellow head virus (YHV), Taura syndrome virus (TSV), Monodon baculovirus (MBV), *P. monodon* densovirus (PemDNV), Infectious hypodermal and hematopoietic necrosis virus (IHHNV), Decapod iridescent virus 1 (DIV1) and *Vibrio parahaemolyticus*. They undergo cold narcosis in ice-cold water for 5 min prior to dissection. The central nervous system (CNS), hepatopancreas (HP), muscle (Mus), ovary and gill were collected. The CNS was further subdivided into the eyestalk (ES), brain (Br), thoracic ventral nerve cord (VNC-TG), and abdominal ventral nerve cord (VNC-AbG). Note that the VNC-TG included commissural ganglion (CoG), subesophageal ganglion (SEG), and thoracic ganglion (TG). Tissue samples were individually immersed in TriPure isolation reagent (Roche, Santa Clara, CA) and stored at -20°C until RNA isolation. All animal experiments were reviewed and approved by the Animal Ethics Committee, Faculty of Science, Mahidol University (MUSC63-019-527).

### Bioinformatics

Nucleotide sequence of *PemVIH* (GenBank accession no. MW847946) was obtained from our transcriptome data prepared from *P. monodon* central nervous tissue (unpublished data). It was identified by BLASTs searching tools and later named *PemVIH* based on its sequence homology and function. A primer pair, PemVIH-01-F and R (**Table 1**), was designed and used to amplify the *PemVIH* transcript by reverse transcription (RT)-PCR. The PCR amplicon was subcloned into pGEM-T Easy (pGEM-T Easy Vector Systems, Promega, Madison, WI) for sequencing. Bioinformatic analysis of *PemVIH* was performed using The European Bioinformatics Institute (EBI) database (https://www.ebi.ac.uk/ena). Open reading frame (ORF) for the prepropeptide was predicted using Open Reading Frame Finder online software (https://www.ncbi.nlm.nih.gov/orffinder). A putative signal peptide region was predicted by SignalP-5.0 online server (http://www.cbs.dtu.dk/services/SignalP). The available amino acid sequences from the CHH superfamily in other crustacean species were searched in GenBank (https://www.ncbi.nlm.nih.gov/genbank) for multiple sequence alignment analysis using The Hidden Markov Model (HMM)-HMM alignment (HHalign) method of Clustal Omega *via* Uniprot website (https://www.uniprot.org/align). Accession numbers and abbreviations of crustacean hormones used for the analyses are shown in **Table 2**. A phylogenetic tree was constructed using the Maximum Likelihood analyses with Molecular Evolutionary Genetics Analysis (MEGA-X) program.

**Table 1:** Lists of specific primers used in this study.

Genes	Primers^1^	Sequence 5’- 3’	Amplicon, bp	Purposes
*PemVIH*	PemVIH-01-F PemVIH-01-R PemVIH-02-F PemVIH-02-R	CGCCACTTCAGTGGGGTCGACATAA GCGTCGAGGTCGCGGCTGACGAATG GACGACGAGTGCGTGGGCGCGATGG TTATTTTCGGCCAGCTTTGAGGATAC	659 228	Gene validation and expression ds*PemVIH* synthesis
*PemVg*	PemVg-F PemVg-R	CTAAGGCAATTATCACTGCTGCT AAGCTTGGCAATGTATTCCTTTT	354	Gene expression
*Actin*	Actin-F Actin-R	CCCAGAGCAAGAGAGGTA GCGTATCCTTCGTAGATGGG	350	Gene expression
*EGFP*	EGFP-F EGFP-R	ATGTCTATGGTGAGCAAGGGCG GCCGTCCTCGATGTTGTGGCGG	531	ds*EGFP* synthesis

^1^F, forward; R, reverse.

**Table 2:** Accession numbers of CHH superfamily members obtained from GenBank database.

Crustacean species (Abbreviation names)	Types of CHH superfamily	
	CHH-type I (GenBank accession no.)	CHH-type II (GenBank accession no.)
*Penaeus monodon* (Pem)	CHH1 (AAQ24525), CHH2 (AAQ24528), CHH3 (AAQ24529)	GIH (ABG33898), MIH1 (AAR89516), MIH2 (AAR89517),
*Penaeus japonicus* (Pej)	CHH (BAE78493)	MIH (BAA20432)
*Litopenaeus vannamei* (Liv)	CHH (AJK31205)	GIH (AHJ11242), MIH1 (ABD73291), MIH2 (ABD73292), VIH (AGX26044)
*Metapenaeus ensis (*Mee*)*	CHH (BAJ23164)	GIH (AAL33882), MIH (AAC27452)
*Homarus gammarus* (Hog)	–	VIH (ABA42181)
*Scylla paramamosain* (Scp)	–	VIH (AHE40786)
*Scylla olivacea* (Sco)	–	VIH (AZF98733)
*Macrobrachium rosenbergii* (Mar)	CHH (AAF29534)	MIH (AGN90993), SGPA (AAL37948), SGPB (AAL37949)
*Macrobrachium nipponense* (Man)	–	GIH (AEJ54623), MIH (AIP90070)
*Penaeus chinensis* (Pec)	–	MIH (AAL55258)
*Penaeus indicus* (Pei)	–	GIH (ALZ42078)
*Penaeus semisulcatus (*Pes)	–	GIH (BAV60264), MIH (BAN05500)

The putative three-dimensional structure of PemVIH was predicted using the SWISS MODEL server (https://www.swissmodel.expasy.org). The server assembled PemVIH by referencing the Protein Data Bank (PDB). Hormone structure was visualized *via* UCSF Chimera 1.13.1 program.

### RT-PCR and Quantitative PCR

To extract RNA, tissue samples were homogenized in TriPure reagent (Roche) and incubated in chloroform for 10 min and centrifuged at 4°C, 13,200 *×g* for 15 min. Supernatant containing total RNA was precipitated using isopropanol, washed twice with ethanol, and dissolved in RNase-free water. RNA samples were quantified using a nanodrop spectrophotometer (ThermoScientific, Waltham, MA), and RNA integrity was assessed by formaldehyde agarose gel electrophoresis. One microgram of RNA was treated with 1U of DNaseI at 37°C for 1 h to eliminate genomic DNA. cDNA was synthesized using a RevertAid First-Strand cDNA synthesis kit (#K1612, ThermoScientific) according to the manufacturer’s instructions and stored at -80°C until use.

RT-PCR was performed using i-Taq Plus DNA Polymerase kit (#25151, INtRON, Gyeonggi-do, South Korea). The PCR cocktail (10X PCR buffer, 0.2 µM/µl, primers mixture, 0.2 mM dNTPs, and 0.05 U/µl of Tag polymerase) was prepared as a master-mix and added batchwise to each cDNA template sample (100 ng). The cDNA template for actin amplification was diluted two times. Specific primers for *PemVIH* and a reference gene are listed in **Table 1**. PCR conditions were 94°C for 15 s, followed by 40 cycles at 94°C for 15s, 58°C for 30s, and 72°C for 40s, and lastly with a final extension step at 72°C for 7min. Negative controls were amplified using sterile water instead of cDNA template under the same RT-PCR protocol. PCR products were subjected to agarose gel electrophoresis and stained with ethidium bromide. PCR products were visualized using Geldoc (Syngene, Frederick, MD).

For quantitative PCR (qPCR), *PemVIH* expression was determined in VNC-TG, whereas *vitellogenin* (*PemVg*) expression was determined in HP and ovary. Primers for *PemVg* were designed according to accession no. EE332453.1 of GenBank database (20). Actin was used as the reference gene. The cDNA template was diluted at 1:4 for *PemVIH* and *PemVg*, and 1:16 for actin amplification. A master mix for qPCR (0.4 μl of primers mix, and 2X master mix of KAPA SYBR buffer (#KK4600, Roche) was prepared, and the assay was performed according to the recommended protocol (Roche).

Relative gene expression was calculated according to Livak and Schmittgen (2001) (21). Differences in relative gene expression between control and experimental groups were analyzed by Student’s *t*-test with α = 0.05. Statistical analysis was performed using Prism version 5 (GraphPad, San Diego, CA). All experiments were performed in triplicates.

### Antibody Production

A rabbit polyclonal antiserum against PemVIH was generated by Genscript Biotech, Piscataway, NJ. Briefly, a synthetic peptide CIMASERHAEVEQFN was designed from a hydrophilic domain of the predicted PemVIH amino acid sequence (87^th^ – 101^st^), conjugated to chicken ovalbumin *via* the cysteine residue, and used for three rounds of immunizations. The resulting serum from the final bleed was collected and used for immunofluorescence study described below.

### Whole Mount Immunofluorescence

To localize PemVIH in shrimp tissues, whole mount immunofluorescence (WM-IF) was performed as described previously by Kruangkum et al. (2013) (22). Dissected shrimp tissues were fixed in 4% paraformaldehyde in 0.1 M phosphate-buffered saline (PBS) for 24 h, then washed in PBS at 4°C. The fixed CNS tissues, including the brain and the entire ventral nerve cord spanning thoracic and abdominal regions, were dissected and immersed in PBS containing 0.2% Triton-X100 (PBST) overnight at 4°C. Tissues were subsequently permeabilized using Dent’s fixative (mixture solution of 80% Ethanol and 20% DMSO) for 8 h at -20°C, washed with cold PBS and PBST at 4°C for 15 min, 3 times each. The CNS tissues were then incubated in the anti-PemVIH polyclonal antiserum (1:500) in a blocking solution (10% normal goat serum in PBST), and gently shaken at 4°C for 5 days. Negative controls included CNS tissues incubated with the anti-PemVIH antiserum preadsorbed with 10 µM immunizing peptide. The tissue samples were then washed separately three times each in PBST and PBS, incubated in an Alexa 488-conjugated goat anti-rabbit antibody (Santa Cruz, Dallas, TX; 1:1,000) and a nuclear staining probe TO-PRO-3 (Invitrogen, Waltham, MA; 1:2,000) in blocking solution at 4°C for 3 days. Afterwards, the specimens were washed 3 times each with PBST and PBS, gradually dehydrated in increasing concentrations of ethanol (50% to 100%), and cleared in methyl salicylate overnight. Tissues were observed and photographed under an inverted Olympus FV1000 confocal microscope.

### *In Vitro* Synthesis of dsRNA Specific to *PemVIH*

dsRNA specific to *PemVIH* (ds*PemVIH*) was synthesized using a bacterial cell culture system previously described (23). Briefly, DNA templates for generating ds*PemVIH* and *EGFP* (green fluorescence protein*)* were amplified with gene specific primers (**Table 1**) using our RT-PCR protocol. PCR amplicons were ligated into pGEM-T Easy vector and pDrive cloning vector, and subjected to sequencing analysis. A sense strand was further digested and recombined into an antisense strand-containing plasmid prior to transformation into *E. coil* (HT115). The dsRNA was isolated from cells using TriPure reagent. Any contaminated DNA and single stranded RNA was eliminated by 1 U of DNaseI and 1 U of RNase A digestion at 37°C for 1 h. The amount and integrity of dsRNA was evaluated by a nanodrop spectrophotometer and agarose gel electrophoresis, respectively.

### Temporal Knockdown of *PemVIH* by RNA Interference and Its Effect on Vitellogenesis

Twenty-four subadult female shrimp were used in an *in vivo* knockdown bioassay. Shrimp were divided into 2 groups (12 shrimp/group): 1) an experimental group treated with ds*PemVIH* (10 μg/g body weight), and 2) a control group treated with ds*EGFP* (10 μg/g body weight). All shrimp were injected intramuscularly once with 80-100 μl of the dsRNA dissolved in PBS, kept in individual tanks for 4 days under the pre-injection environment, and fed commercial food twice a day. Afterwards, all shrimp were sacrificed for tissue collection. Since vitellogenesis takes place especially in ovary and hepatopancreas (HP), *PemVg* expression was determined in both ovary and HP. The relative expression of *PemVIH* and *PemVg* was evaluated by qPCR as described above.

## Results

### PemVIH Exhibits Conserved Features of Crustacean Hyperglycemic Hormone-Type II

**Figure 1:**
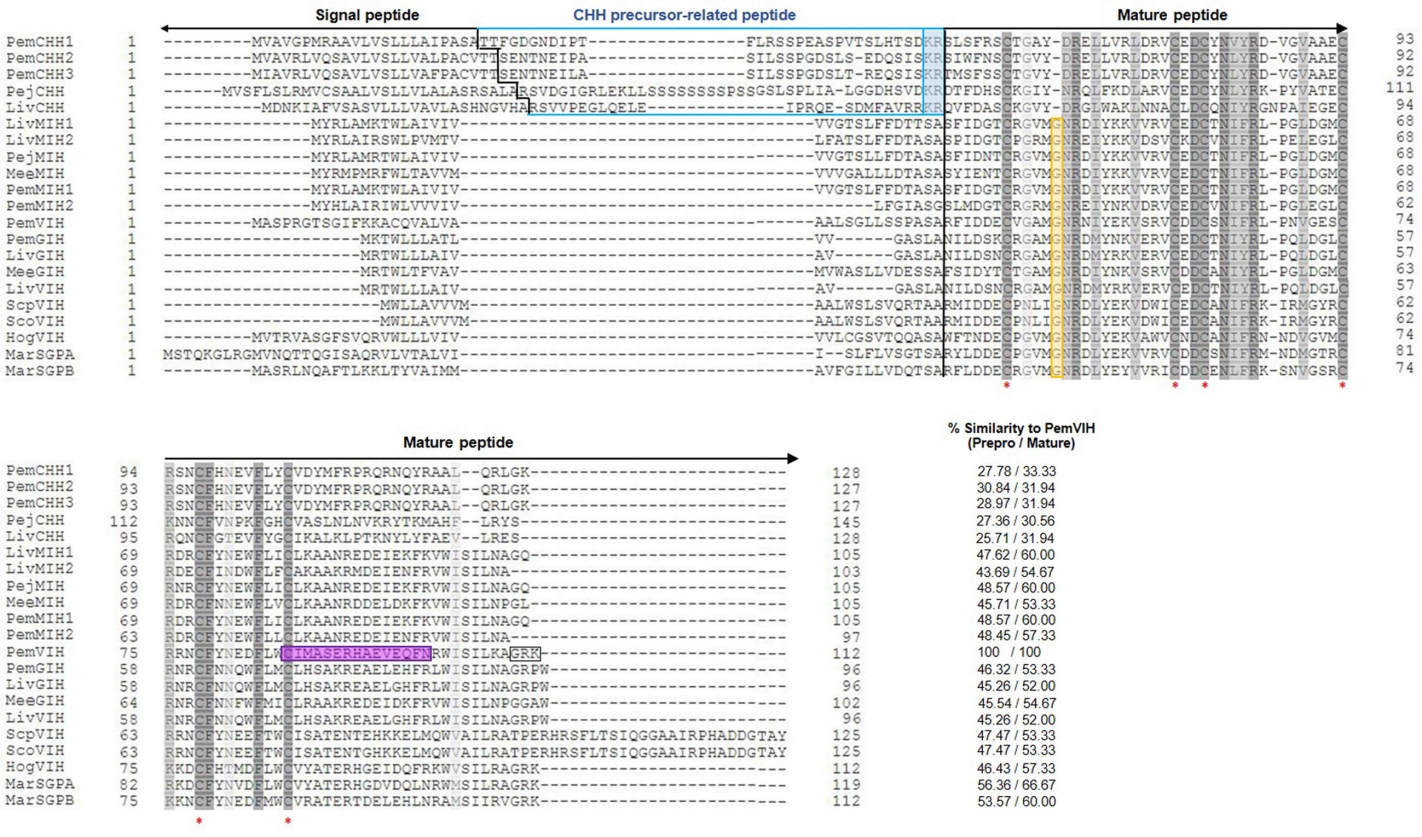
Multiple amino acid sequence alignment of CHH superfamily in crustaceans. It includes PemVIH, PemCHH1, PemCHH2, PemCHH3, PejCHH, LivCHH, LivMIH1, LivMIH2, PejMIH, MeeMIH, PemMIH1, PemMIH2, PemGIH, LivGIH, LivVIH, MeeGIH, ScpVIH, ScoVIH, HogVIH, MarSGPA, and MarSGPB (see species of origin and accession numbers in **Table 2**). Amino acid numbering is indicated in margins of the sequences. Percent similarity to the prepro- and mature PemVIH is shown in the bottom right column. Identical and similar amino acid residues are shaded in dark gray and light gray, respectively. Red asterisks indicate six conserved cysteine residues. A black vertical line indicates the predicted signal peptide cleavage sites. A blue line covers CPRP of CHH-type I and blue highlight shows dibasic residue (KR) of CHH-type I. The conserved glycine residues of CHH-type II are highlighted in yellow. The peptide sequence that was selected for antibody production against PemVIH is highlighted in purple. Boxed area indicates the predicted dibasic cleavage site of PemVIH at “RK” and amidation site at “G”.

A full-length 1,022 bp cDNA of *PemVIH* was identified in transcriptomic data of *P. monodon* CNS. The nucleotide sequence consisted of 275 bp of 5′untranslated region (UTR), 339 bp of ORF and 408 bp of 3′ UTR. The ORF encoded a peptide of 112 amino acids with a conserved domain exhibiting significant sequence similarity to other CHH superfamily members (**Figure 1**). A signal peptide prediction showed a significant cleavage position at Ala^34^, and a proposed dibasic cleavage site at Arg^111^-Lys^112^ with amidation site at Gly^110^giving rise to a mature peptide of 75 amino acids with a calculated molecular mass of 8.85 kDa (see **Supplement 1**).

Multiple alignment classified the putative PemVIH as a member of the CHH superfamily based on the presence of six highly conserved cysteine residues (Cys^41^, Cys^58^, Cys^61^, Cys^74^, Cys^78^ and Cys^87^) characteristic of this superfamily. Moreover, CHH precursor-related peptide (CPRP), a characteristic of CHH-type I family, was not found in PemVIH. Instead, it contained a glycine residue (Gly^46^) 5 amino acids downstream of the first cysteine residue (Cys^41^), indicating that the PemVIH belonged to the CHH-type II family (**Figure 1**).

**Figure 2:**
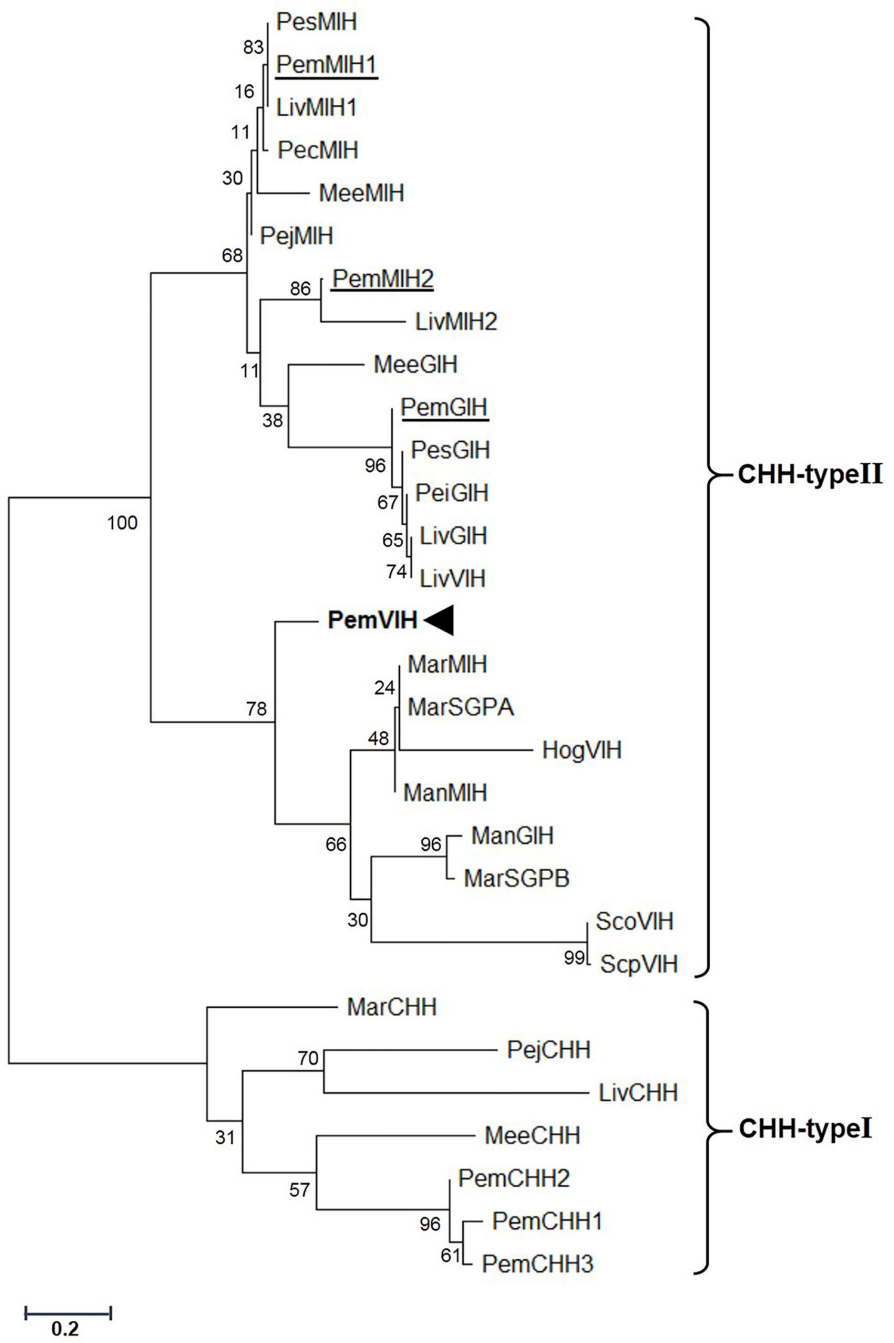
Phylogenetic analysis of PemVIH and other crustacean CHH superfamily members. The number of each node showed bootstrap values generated by the Maximum Likelihood method (1,000 replication). Species of origin for CHH-type I and CHH-type II are included in the abbreviated names (see **Table 2**) on the right side. PemVIH is indicated in bold, whereas other CHH-type IIs in *P. monodon* are underlined.

Percent similarity of PemVIH with other crustacean hormones is shown in **Figure 1**. The putative mature peptide of PemVIH showed the highest percent similarity to *Macrobrachium rosenbergii* sinus gland peptide A (*MarSGPA*) *at* 66.67%. Focusing on hormones in Penaeid shrimp, PemVIH showed the highest percent similarity to *Peneaus japonicus* molt-inhibiting hormone (PejMIH) and *P. monodon* molt-inhibiting hormone 1 (PemMIH1) at 60%, followed by PemMIH 2 and *P. monodon* gonad-inhibiting hormone (PemGIH) at 57.33% and 53.33%, respectively. A maximum likelihood phylogenetic analysis also grouped PemVIH into the CHH-type II family (bootstrap value 100%) and suggested a monophyletic clade of PemVIH in relation to other VIHs (bootstrap value 78%) (**Figure 2**). This suggested that PemVIH is a novel *P. monodon* CHH-type II.

**Figure 3:**
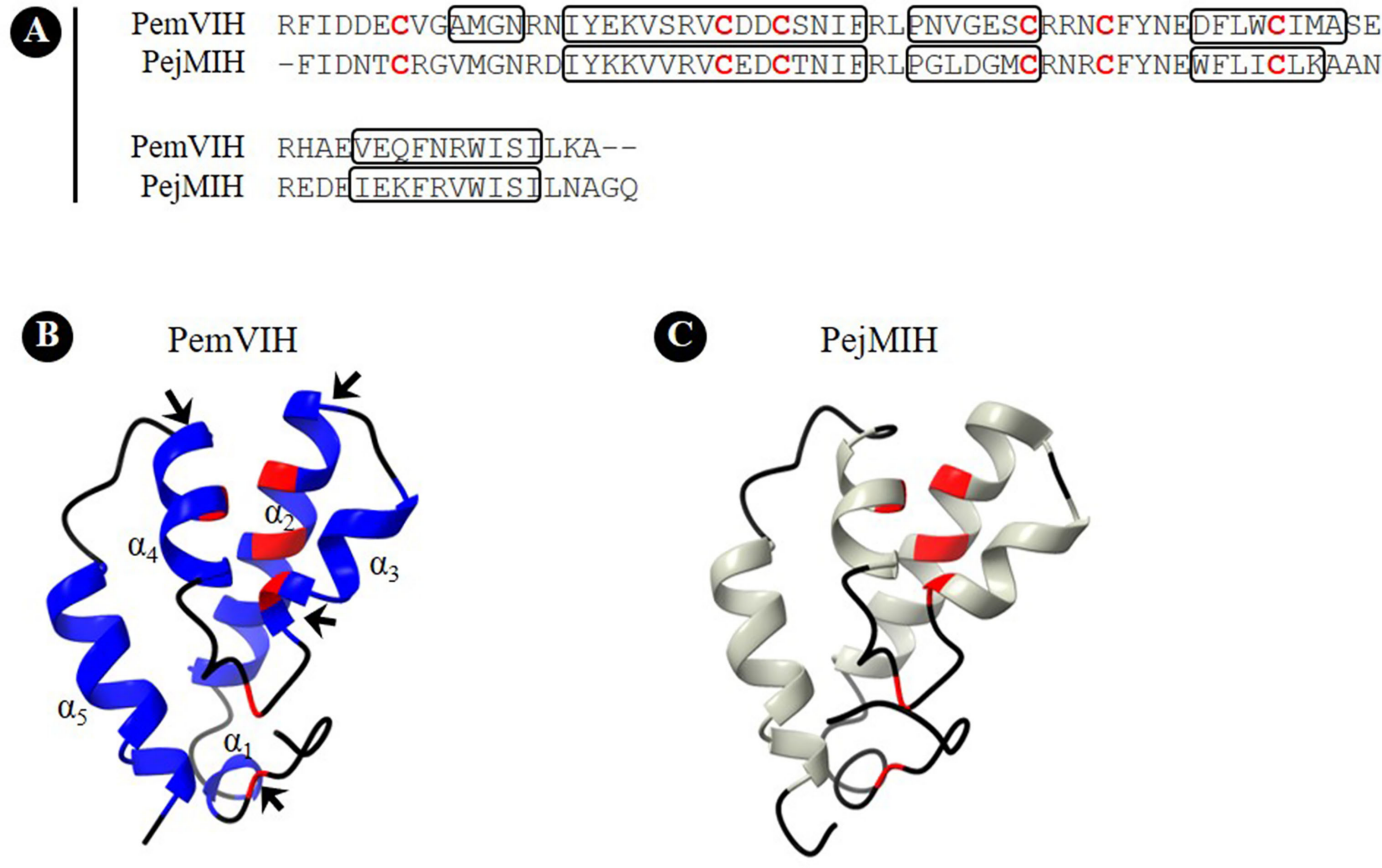
Putative three-dimensional structure of PemVIH. **(A)** Amino acid sequence alignment of PemVIH and PejMIH. Conserved cysteine residues are shown in red alphabet. Boxed area indicates loop structure. Putative three-dimensional structure of **(B)** PemVIH and **(C)** PejMIH consist of 5 and 4 α-helices, respectively. The conserved six cysteine residues are labelled in red. Structural differences between PemVIH and PejMIH are indicated by the arrows in **(B)**.

A putative three-dimensional structure of PemVIH was constructed and compared against PejMIH. As a member of CHH-type II, the conserved six cysteine residues were shown in both hormones at position 8^th^, 25^th^, 28^th^, 41^st^, 45^th^ and 54^th^ (**Figure 3A**). However, based on the PDB ID (code 1jot) and our sequence, the putative structure of PemVIH had five predicted α-helices (**Figure 3B**), whereas PejMIH only had four (**Figure 3C**). The lengths of α-helices were also different between the two peptides. The α_2_-helix of PemVIH was shorter than that of PejMIH, whereas α_3_- and α_4_-helices of PemVIH were extended compared to PejMIH.

In addition, the three-dimensional structures of *P. monodon* CHH-type II family, including PemGIH, PemMIH1 and PemMIH2, were constructed and compared with PemVIH. All of them showed five α-helices and the root-mean square distance (RMSD) values between them were ~ 2 Å suggesting structural similarity of the *P. monodon* CHH-type II (see **Supplement 2**). Moreover, the putative surface structure of PemVIH and PemGIH was compared. Area comparison of acidic, basic, and hydrophobic residues in the two peptides and electrostatic potential mapped onto the surface area was demonstrated (see **Supplement 3**). The surface representations showed the similar surface area properties of the two peptides.

### *PemVIH* Transcript Was Specifically Detected in Brain and Ventral Nerve Cord, Not The Eyestalk

**Figure 4:**
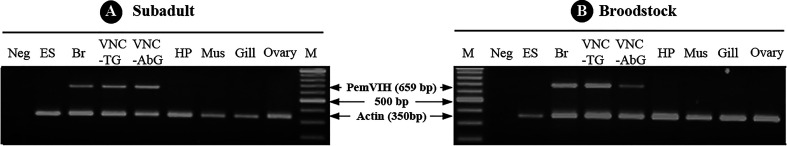
*PemVIH* expression profile. RT-PCR of *PemVIH* in **(A)** subadult and **(B)** broodstock female *P. monodon*. The *PemVIH* and *actin* amplicons are 659 bp and 350 bp, respectively. *PemVIH* was specifically expressed in the nervous tissues including Br, VNC-TG and VNC-AbG of subadult and broodstock, but not the ES. M, DNA ladder; Neg, negative control.

Expression of *PemVIH* transcript was determined in CNS and non-CNS tissues of female subadults and broodstock. The CNS tissues included ES, Br, VNC-TG, and VNC-AbG, whereas the non-CNS tissues included HP, Mus, ovary, and gill. RT-PCR revealed the expression of *PemVIH* transcript as a 659-bp amplicon only in the Br, VNC-TG, and VNC-AbG, but not in the ES and non-CNS tissues of both female subadult (**Figure 4A**) and broodstock shrimp (**Figure 4B**).

### PemVIH Was Intensely Localized in Neurons of Commissural Ganglia

**Figure 5:**
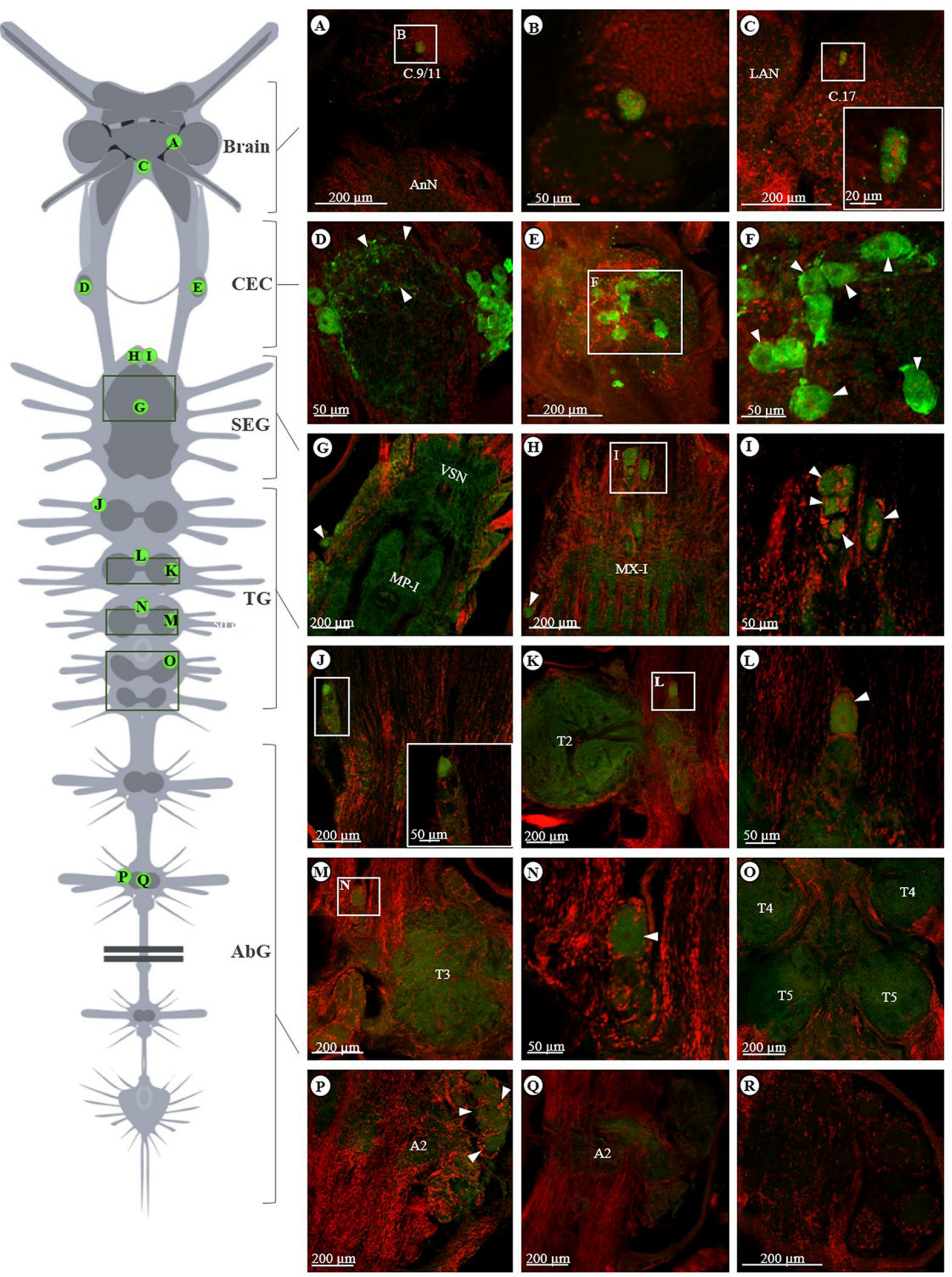
Localization of PemVIH in nervous tissues of subadult female *P. monodon*. A schematic diagram on left illustrates shrimp’s central nervous system including brain, CEC, SEG, TG and AbG. Green circles with letters indicate distribution of PemVIH signals in regions corresponding to Panels **(A–R)**. Green fluorescence represents positive PemVIH-ir, whereas red fluorescence indicates nuclear specific staining of TO-PRO3. **(A, B)** PemVIH-ir was observed in small neurons in neuronal cluster 9/11 (magnified in **B**) and **(C)** medium size neurons in cluster 17 of the brain. Intense PemVIH-ir was detected in the **(D)** neuropils (arrow heads) and **(E, F)** neurons of CEC (magnified in **F**). Moderately intense signal of PemVIH-ir was detected in **(G–I)** neurons (arrowheads) and **(G, H)** neuropils of SEG and **(J–O)** TG. **(P, Q)** A weak signal of PemVIH-ir was observed in AbG. **(R)** The negative control where the representative of CEC tissue incubated with PemVIH peptide-preadsorbed antiserum showed no PemVIH-ir. MP-I, maxilliped neuropil-I; MX-I, maxillary neuropil-I; VSN, ventral sensory neuropil.

Localization of PemVIH was elucidated using WM-IF staining. PemVIH immunoreactivity (-ir) was detected in CNS tissues including the Br, commissural ganglion (CoG), subesophageal ganglion (SEG), thoracic ganglion (TG) and abdominal ganglion (AbG) (**Figure 5**, left panels).

In the brain, PemVIH-ir was observed in neuronal cluster number 9/11 of deuterocerebrum (**Figures 5A, B**) and neurons in cluster 17 of tritocerebrum (**Figure 5C**, magnified in the inset). Surprisingly, the intensive PemVIH-ir was detected in a pair of CoG, a component of circumesophageal commissures (CEC). The fluorescent signal was present not only in nerve fibers linking medial and lateral neurons (**Figure 5D**, arrowheads), but also localized in the medial and lateral neurons of the CoG (**Figures 5E, F**, arrowheads).

In additon, PemVIH-ir was shown in neuropils of subesophageal ganglion (SEG) (**Figures 5G, H**). It was found in the dorsolateral cluster (DLC) (**Figures 5G, H**, arrowheads) and ventromedial cluster (VMC) of visceral sensory neuropils (VSN) (**Figure 5I**, arrowheads). More posteriorly, PemVIH-ir was present in the DLC of the 1^st^ TG (**Figure 5J**) and scattered in the neuropils of ventral ganglia including TG (**Figures 5K, M, O**). PemVIH-ir was also found in the VMC of 2^nd^ and 3^rd^ TG, as shown in **Figures 5L, N**, respectively. Lastly, diffused PemVIH-ir was found in the DLC (**Figure 5P**, arrowheads) and neuropils of AbG (**Figure 5Q**). The negative control using the preadsorbed antiserum showed no immunoreactivity (**Figure 5R**).

### Temporal Knockdown of *PemVIH* Increased Vitellogenesis in Ovary

**Figure 6:**
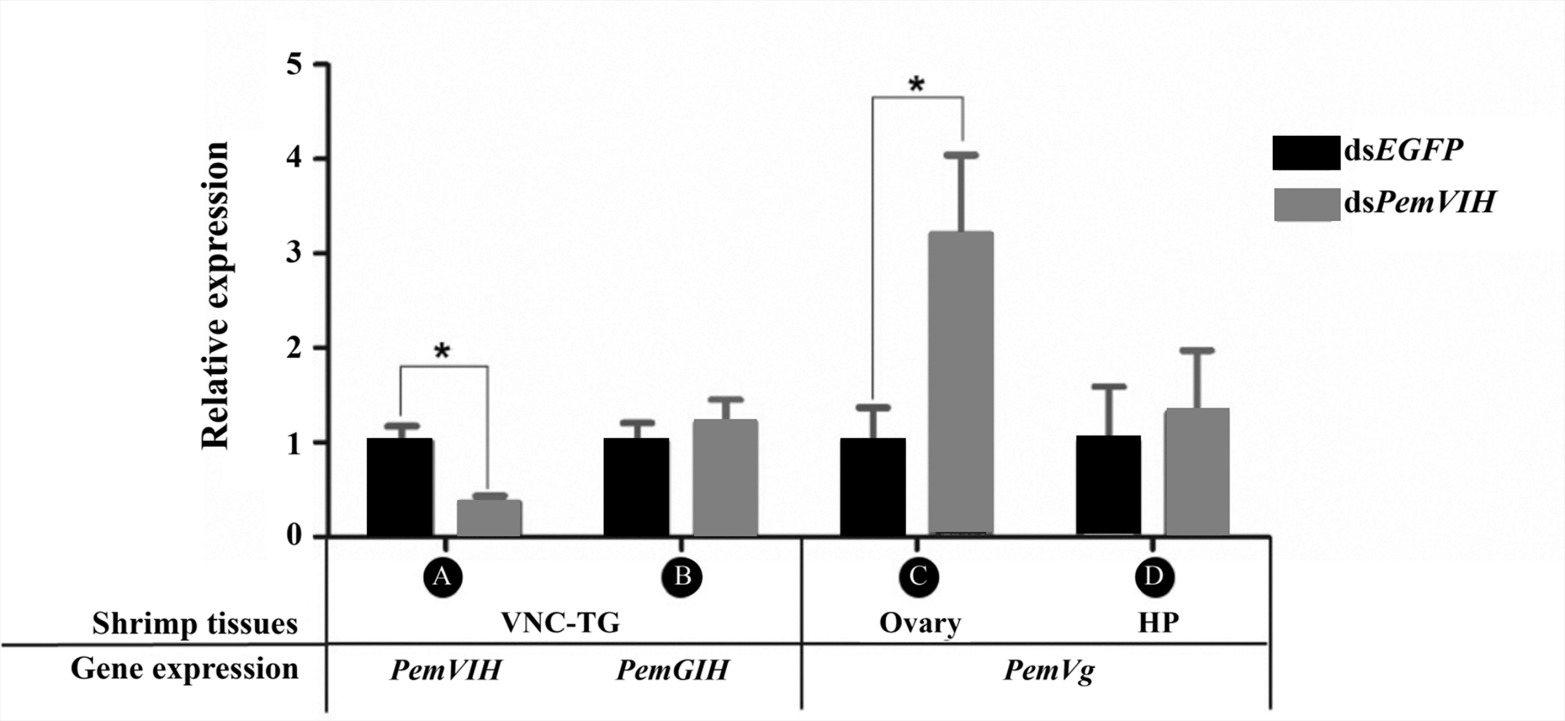
Efficiency of ds*PemVIH* on *PemVIH* suppression and its effect on vitellogenesis. The relative expression of **(A)** *PemVIH* and **(B)** *PemGIH in* ds*PemVIH* treated group compared with the ds*EGFP* treated group. *PemVIH* but not *PemGIH*, was significantly decreased. The relative expression level of *PemVg* in **(C)** ovary, but not **(D)** HP was significantly increased in ds*PemVIH*-injected group compared with the ds*EGFP*-injected group. Each bar = mean ± SEM (n = 10). Asterisks represented significant difference at *P ≤ 0.01.

**Figure 7:**
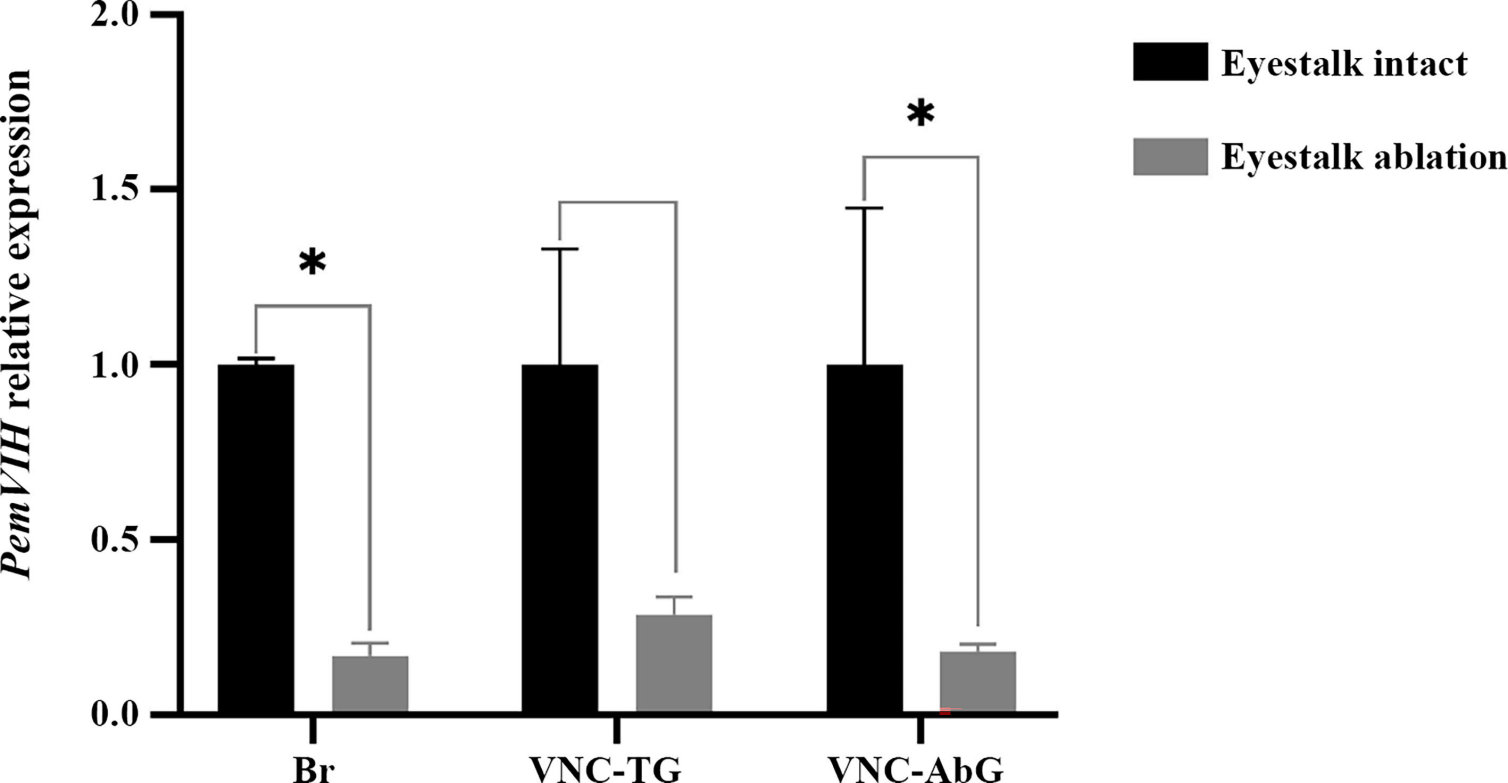
Effect of eyestalk ablation on *PemVIH* expression. Relative expression of *PemVIH* (*PemVIH/actin*) was significantly decreased in Br and VNC-AbG of eyestalk-ablated females. Note that the VNC-TG included commissural ganglion (CoG), subesophageal ganglion (SEG), and thoracic ganglion (TG). Each bar = mean ± SEM (n = 3). Asterisks indicated statistic difference with *P < 0.05.

To determine the effect of *PemVIH* on vitellogenesis, a single dose of ds*PemVIH* was injected into female subadults at a concentration of 10 μg/g body weight to suppress the expression of *PemVIH*. We first evaluated the efficiency and specificity of the synthetic dsRNA administration on *PemVIH* suppression in VNC-TG. The expression of both *PemVIH* and *PemGIH* was determined after ds*PemVIH* and ds*EGFP* administration. *PemVIH* was significantly downregulated in the ds*PemVIH*-treated group compared to the ds*EGFP*-treated group (**Figure 6A**). In contrast, *PemGIH* expression level was not significantly different between ds*PemVIH-* and ds*EGFP*-treated groups (**Figure 6B**). Since both HP and ovary produced vitellogenin, the expression of *PemVg* was determined in both tissues after ds*PemVIH* and ds*EGFP* administrations. Results showed that the expression of *PemVg* in HP was not significantly different between both groups (**Figure 6C**). However, in the ovary, the expression of *PemVg* was significantly upregulated by three-fold in the ds*PemVIH-*injected group compared to the ds*EGFP-*injected group (**Figure 6D**). In addition, an effect of eyestalk ablation on *PemVIH* expression was evaluated in broodstocks. A significant decrease of *PemVIH* expression level was shown in Br and VNC-AbG of the eyestalk-ablated group (**Figure 7**). However, in VNC-TG, *PemVIH* expression level was lower in the eyestalk-ablated group, but it was not statistically significantly different.

## Discussion

CHHs are neuropeptide hormones synthesized mainly in the Penaeid shrimp CNS and X-organ sinus gland complex of the eyestalks (24–27). They perform diverse functions including metabolic and reproductive controls (25, 28). CHHs can be categorized into 2 types, CHH-type I and CHH-type II, based on their sequences (29). The CHH-type I has been reported in several species, including a crab (*Callinectes sapidus*), a crayfish (*Procambarus clarkii*), and several shrimp species (30–32). They exhibit diverse functions in glucose metabolism, osmoregulation, and environmental-stress responses (33, 34). In contrast, the CHH-type II has been more narrowly characterized as inhibitory hormones affecting molting and reproductive functions (35–37). In *P. monodon*, up to five CHH-type II, including PemC1-2 (38), PemMIH1-2 (39) and PemGIH (20), have been identified in the eyestalk. In this study, a novel CHH-type II, namely PemVIH, was identified and characterized for its regulation, production sites and biological activities.

Analysis of PemVIH cDNA sequence revealed a mature peptide with features consistent with the CHH-type II family, such as six highly conserved cysteine residues, a glycine residue 5 amino acids downstream of the first cysteine residue, and the absence of the CPRP domain (19). Generally, CHH-type II members consist of 78-83 amino acids with a molecular weight of approximately 8-9 kDa (20). Multiple alignment showed greatest sequence similarity between PemVIH and *MarSGPA*, the latter of which belongs to MIH/VIH group of CHH-type II (40). Phylogenetic analysis clustered PemVIH into a unique CHH-type II clade distinct from other identified *P. monodon* CHH-type II hormones (**Figure 2**), suggesting PemVIH is a novel *P. monodon* CHH-type II. Although the tertiary structure of a hormone is important for receptor binding specificity, putative three-dimensional structures of the CHH superfamily members were rarely reported (41, 42). Note that the structure of PejMIH reported by Katayama et al., 2003 has five helices (41); however, our study showed only four helices, probably due to different analysis methods. Our study comparing the putative structures of *P. monodon* CHH-type II and PemVIH demonstrated the structural similarity of PemVIH and other reported *P. monodon* CHH-type II (see **Supplement 2**). Moreover, the putative surface representations of PemVIH and PemGIH showed certain similar surface area properties (see **Supplement 3**), suggesting they interact with very similar receptors. It has been shown that CHH peptide hormones commonly bind with G protein-coupled receptors (GPCRs) on target cells (43, 44). In *L. vannamei* and *C. sapidus*, LivMIH2 and CasMIH have been shown to regulate vitellogenesis in addition to their role in ecdysis (28, 37). This could be due to the similar structure of hormones that could bind to the similar receptor. However, the role of PemMIH1 and PemMIH2 in regulating *P. monodon* vitellogenesis have never been reported.

In crustaceans, VIH is mainly synthesized and stored in X-organ/sinus gland (XO/SG) complex in eyestalks (13, 45, 46). However, the brain and ventral nerve cord have also been reported as a major source of VIH in some species, including *L. vannamei* (17), and the mud crab *Scylla olivacea* (13). In this study, we showed, by RT-PCR, that the newly reported *PemVIH* was not expressed in the eyestalk of *P. monodon.* Instead, *PemVIH* was expressed in the brain and ventral nerve cord of subadult and broodstock female shrimp (**Figure 4**). Interestingly, although *PemVIH* was not expressed in eyestalks, eyestalk ablation significantly downregulated *PemVIH* expression especially in Br and VNC-AbG (**Figure 7**). This suggested that eyestalks may contain an upstream regulator of *PemVIH*; this possibility remains to be investigated.

Since *PemVIH* was expressed only in the central nervous tissues, immunolocalization of PemVIH focused exclusively on the CNS and ventral nerve cord. An antiserum was generated against a synthetic peptide mapped to a hydrophilic and unique sequence of PemVIH (47). WM-IF revealed PemVIH-ir in neuronal cluster 9/11 and 17 of brain, commissural ganglion (CoG), subesophageal ganglion (SEG), thoracic ganglion (TG) and abdominal ganglion (AbG) (**Figure 5**). Our results are consistent with the recent study of ScoVIH in the mud crab demonstrating the localization of *ScoVIH* transcript in clusters 6, 11 of the brain and in neurons of SEG and TG by *in situ* hybridization (13). Interestingly, PemVIH-ir was intensively localized in CoG. CoG is a part of the stomatogastric nervous system (STNS) that regulates movement of the digestive tract (48, 49). In the lobster *Homarus gammarus*, CoG consists of many peptidergic neurons that secrete C-type allatostatin (50). In marine crab *Cancer productus* and *Cancer borealis*, CoG is also a neuroendocrine organ secreting tachykinin-related peptide Ia (51), histamine-like peptide (52), and red pigment-concentrating hormone (53). A relationship between CoG and CHH superfamily has never been described before, thus our study is the first to report the presence of CHH-type II in CoG of a crustacean. Our findings showed that PemVIH was strongly localized in both medial and lateral neurons of the CoG, suggesting that CoG was the main source of PemVIH and may release it into the circulation to act as a bona fide hormone. In support of this notion, hemolymph sinuses have been shown within the CoG of *C. productus* (51). The presence of PemVIH in CoG, a part of STNS, also suggests it may mediate the crosstalk between nutritional and reproductive signaling. For example, it may play a role in the previously reported stimulation of vitellogenesis by feeding and inhibition of vitellogenesis by starvation (54, 55). In contrast, PemGIH is localized in the sinus gland and eyestalk ganglia of broodstock females (27). Even though the *PemGIH* transcript is also demonstrated in the brain and ventral nerve cord, cellular localization of PemGIH peptide has not been revealed in such tissues. Dynamic expression and localization of *PemVIH* and its coordination with *PemGIH* expression during ovarian development remain to be addressed.

In crustaceans, VIH is known to suppress vitellogenesis (14, 19, 56). RNAi knockdown strategy has been conventionally employed to understand the physiological role of a hormone (20, 57–59). The study of Treerattrakool et al. (2008) (20) showed that PemGIH knockdown can inhibit ovarian vitellogenesis in *P. monodon* broodstocks. For this study, we used a dsRNA knockdown strategy previously proven to be successful in suppressing *PemGIH* (20). We showed a single injection of ds*PemVIH* was highly effective in downregulating the expression of *PemVIH*, but not *PemGIH*, in subadults (**Figure 6**). This downregulation of *PemVIH* was associated with the upregulation of *PemVg* in the ovary, but not HP (**Figure 6**). Our result suggested that *PemVIH* could regulate ovarian vitellogenesis in subadult females. In *P. monodon*, early expression of *PemVg* could be detected in the premature ovary of juvenile shrimp and presented throughout the ovarian maturation, especially in the vitellogenic stage (60). Moreover, a certain amount of Vg protein could also be detected in hemolymph of subadult shrimp, however, the levels were lower than in adults (27, 61). Hepatopancreas and ovary have been known as the site of vitellogenesis (62–64), and multiple Vg genes have been reported in some species, including *Pandalopsis japonica* (65), *Metapenaeus ensis* (66) and *L. vannamei* (67). In *M. ensis*, expression level of *MeVg1* was relatively high in HP but low in ovary (66). In contrast, in *P. monodon*, *PemVg* expression in ovary was as high as HP during ovarian maturation (63), suggesting that both organs contributed equally to vitellogenesis. It is possible that our *PemVIH* knockdown failed to affect HP *PemVg* due to the short-term nature of dsRNA administration, or there may be different hormones regulating tissue-specific vitellogenesis. In addition, whether the ovarian vitellogenesis is dominant in the initial cycle of ovarian maturation in subadult *P. monodon* remains to be investigated.

In *P. monodon*, knockdown of *PemGIH* by dsRNA resulted in the enhancement of ovarian vitellogenesis (20). In addition, suppression of PemGIH by RNA interference (RNAi) and antibody neutralization led to an increase in percent ovarian maturation and spawning of female broodstock (7, 8). However, RNAi and antibody neutralization combined was still less effective than unilateral eyestalk ablation in accelerating ovarian maturation and spawning. This is likely due to multiple hormonal factors that participate in the regulation of ovarian maturation. Our novel *PemVIH* reported herein clearly plays a role in the inhibition of vitellogenesis and could be a synergistic factor of *PemGIH* in regulating vitellogenesis and ovarian maturation in *P. monodon*. An exciting possibility is that the dual suppression of both *PemGIH and PemVIH*, together with a reproductive activator such as 5-hydroxytryptamine (5-HT) (68), could induce ovarian maturation in *P. monodon* as effectively as eyestalk ablation. Overall, our study provided insight into crustacean neurohormones controlling ovarian functions and suggested additional strategies for the endocrine manipulation of female reproduction.

## Data Availability Statement

The datasets presented in this study can be found in online repositories. The names of the repository/repositories and accession number(s) can be found in the article/**Supplementary Material**.

## Ethics Statement

The animal study was reviewed and approved by the Animal Ethics Committee, Faculty of Science, Mahidol University (MUSC63-019-527).

## Author Contributions

PL performed bioinformatic analyses and bioassay, and wrote the first draft of the manuscript. TK and PT performed immunohistochemistry analysis and wrote sections of the manuscript. CC and WS performed molecular analyses and wrote sections of the manuscript. PST contributed to antibody design and production, and critical revision of the manuscript. PS contributed to the conception of the study and critical revision of the manuscript. RV contributed to conception and design of the study and final approval of the version to be published. All authors contributed to the article and approved the submitted version.

## Funding

This research project is supported by Mahidol University under the Fundamental Fund: Basic Research Fund (Fiscal year 2021) to RV and Science Achievement Scholarship of Thailand (SAST) to PL.

## Conflict of Interest

The authors declare that the research was conducted in the absence of any commercial or financial relationships that could be construed as a potential conflict of interest.

## Publisher’s Note

All claims expressed in this article are solely those of the authors and do not necessarily represent those of their affiliated organizations, or those of the publisher, the editors and the reviewers. Any product that may be evaluated in this article, or claim that may be made by its manufacturer, is not guaranteed or endorsed by the publisher.
