# Supplementary material for: Suppression of a Novel Vitellogenesis-Inhibiting Hormone Significantly Increases Ovarian Vitellogenesis in the Black Tiger Shrimp, Penaeus monodon
Source: Front Endocrinol (Lausanne). 2021 Nov 8;12:760538. doi: 10.3389/fendo.2021.760538 (PMC8634883; doi:10.3389/fendo.2021.760538)
Supplement: Supplementary file 1 [file DataSheet_1.pdf]

Supplementary Material

Supplementary Figure 1

```
1    cacgcaacgagagtgacccccctgccatcccccgaagggcaactaacttcttccaggcgc
60   cgcctcctggaccctcgcgcactctctggcccctcccacaccacggccgccgtgtccttg
120  tccttcgccacttcagtggggtcgacataacccttcctggcgcacgtcccggcgcattgc
180  gagacggccgagcagcggtccttcgaacggggcatgcggatagtaccctccagtagtcag
240  gacaccgggcatttttctcccaagacgacccgagtgATGGCATCTCCTCGTGGAACCTCA
1                                        M  A  S  P  R  G  T  S
300  GGCATCTTCAAAAAGGCGTGCCAGGTAGCGTTGGTGGCAGCGGCGTTGTCCGGCCTCCTG
9    G  I  F  K  K  A  C  Q  V  A  L  V  A  A  A  L  S  G  L  L
360  AGCTCGCCCGCCTCCGCCAGGTTCATCGACGACGAGTGCGTGGGCGCGATGGGCAACCGG
29   S  S  P  A  S  A  R  F  I  D  D  E  C  V  G  A  M  G  N  R
420  AACATCTACGAGAAGGTGTCGCGCGTCTGCGACGACTGCAGCAACATCTTCCGCCTTCCG
49   N  I  Y  E  K  V  S  R  V  C  D  D  C  S  N  I  F  R  L  P
480  AACGTGGGCGAGAGCTGTCGGAGAAACTGCTTCTACAACGAGGACTTCCTGTGGTGCATC
69   N  V  G  E  S  C  R  R  N  C  F  Y  N  E  D  F  L  W  C  I
540  ATGGCCTCGGAGCGGCACGCGGAGGTGGAGCAGTTCAACAGGTGGATCAGTATCCTCAAA
89   M  A  S  E  R  H  A  E  V  E  Q  F  N  R  W  I  S  I  L  K
600  GCTGGCCGAAAATAAgacgcacgtcaagtaattccctgtcgtgtatccagcagcctaatg
109  A [G  R  K] *
660  ctacgagtctctcctcctcctgctcctccttcgccttctcccgcgcccttcccttcgccg
720  ccgccactcacgaagtaggacggacgacgcccacgcagacattcgtcagcccgcgacctc
780  gacgccgcccgccaccggatgctgttattgtttcgagtgtgatcgatggcatccccgtcc
840  agcatcgtcagacaaacgtttgcagtctgttactgagcaaattgtcctctaagccgattt
900  tgattcgattcgtcgacgttcagttttcctttattttctcaagggaccacttttagacaa
960  gtcattattatccctaagcacaatgttcacagtacaggcacctttacttttgctttctca
1020 ttg
```

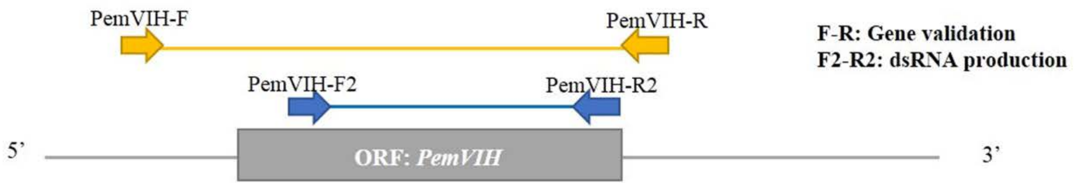

Supplementary Figure 1 (A) Nucleotide and putative amino acid sequences of PemVIH. Numbering of nucleotides and amino acids is indicated in the left margin. Signal peptide is underlined. Yellow- and blue- shaded area indicate the sites of primer designed for gene validation and dsRNA production, respectively. An asterisk indicates the termination codon. Boxed area indicates the predicted dibasic cleavage site at “RK” and amidation site at “G”. **(B)** Diagram showing the sites of primers designed for dsRNA production (blue) and gene validation (yellow).

Supplementary Figure 2

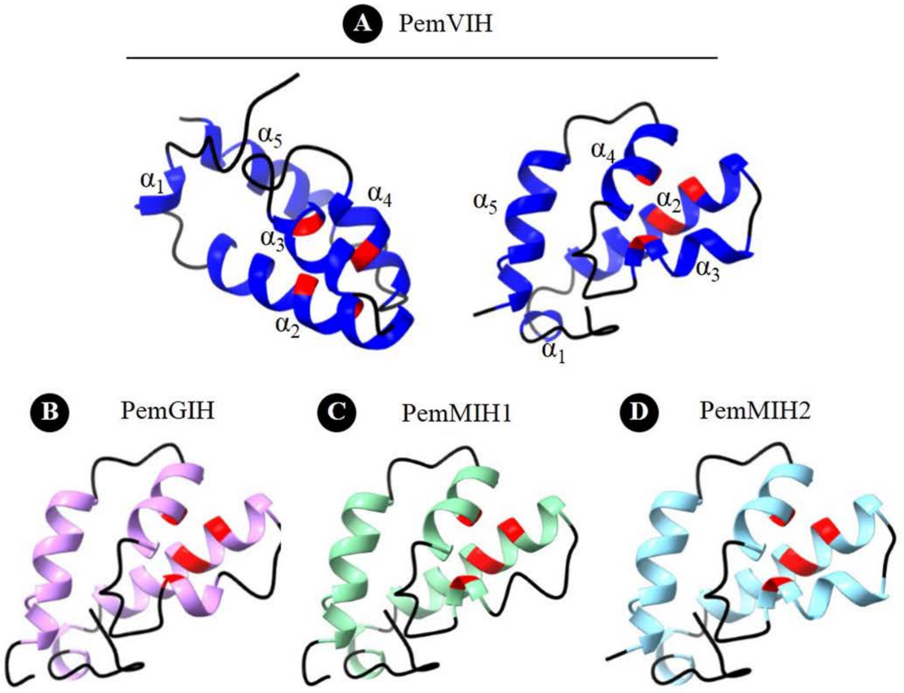

Supplementary Figure 2 Comparative putative three-dimensional structure of *P. monodon's* CHH-type II. Putative three-dimensional structures of *P. monodon*'s CHH-type II, including **(A)** PemVIH, **(B)** PemGIH, **(C)** PemMIH1, and **(D)** PemMIH2, are compared. The root-mean square distance (RMSD) values between all of them are ~ 2 Å suggesting the structural similarity of *P. monodon*'s CHH-type II.

Supplementary Figure 3

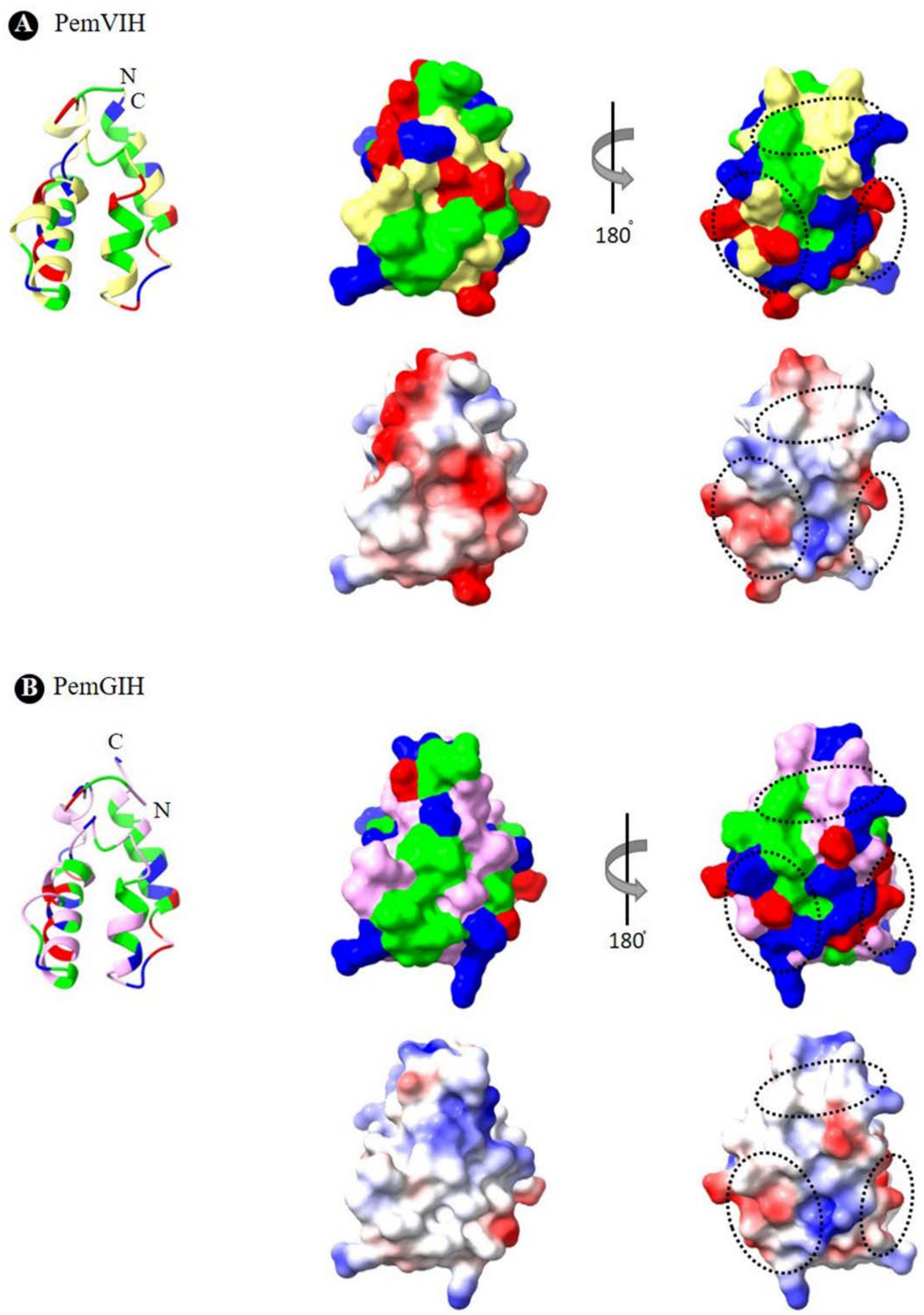

Supplementary Figure 3 Putative surface structure of PemVIH (A) and PemGIH (B). The predicted protein backbone (ribbon model) is shown on the left panel. Upper panels show area comparison of acidic, basic and hydrophobic residues. Lower panels show electrostatic potential mapped onto the surface area of the two peptides. Regions of negative potential are red, those of positive potential are blue, and the neutral regions are white. Circles indicate the similar surface area properties of the two peptides. Results are visualized using UCSF Chimera 1.13.1 program.
